# Prospective multicenter study on the reproducibility of ultrasound-derived fat fraction in assessing hepatic steatosis

**DOI:** 10.1186/s13244-025-02076-5

**Published:** 2025-11-04

**Authors:** Haohao Yin, Guangwen Chen, Yunling Fan, Jifeng Yu, Lin Chen, Hong Han, Liyun Xue, Hong Ding, Huixiong Xu, Yuli Zhu

**Affiliations:** 1 Department of Ultrasound, Zhongshan Hospital, Institute of Ultrasound in Medicine and Engineering, Fudan University, Shanghai, China; 2 Shanghai Institute of Medical Imaging, Fudan University, Shanghai, China; 3 Department of Ultrasound, Huashan Hospital, Fudan University, Shanghai, China; 4 Department of Ultrasound, Huadong Hospital, Fudan University, Shanghai, China

**Keywords:** Nonalcoholic fatty liver disease, Ultrasonography, Ultrasound-derived fat fraction, Reproducibility of results

## Abstract

**Objective:**

This study aimed to investigate the reproducibility of ultrasound-derived fat fraction (UDFF) among operators with different experience levels. Furthermore, it seeks to validate the performance of UDFF in detecting hepatic steatosis.

**Materials and methods:**

The study was conducted at three hospitals and involved patients suspected of having metabolic dysfunction-associated steatotic liver disease (MASLD). Two radiologists took UDFF measurements from each participant to find the best place to measure and evaluate reproducibility. Subsequently, the performance of the UDFF measurements from the left and right liver lobes in detecting hepatic steatosis was compared with magnetic resonance proton density fat fraction (MRI-PDFF) results.

**Results:**

A total of 163 patients were examined for UDFF in both left and right liver lobes. The measurement failure rates were 28.8% for the left lobe and 6.5% for the right lobe. The inter-observer reproducibility was high in the right lobe, with intraclass correlation coefficient (ICCs) of 0.89–0.96. Additionally, 80 participants underwent magnetic MRI-PDFF and UDFF examinations, revealing an area under the curve (AUC) of 0.97 for the right lobe and 0.84 for the left lobe.

**Conclusion:**

UDFF measurements in the left lobe exhibited a higher failure rate and less consistency, while measurements in the right lobe had a higher success rate and excellent reproducibility. For suspected MASLD patients, at least two measurements were recommended. Additionally, UDFF from the right lobe is more reliable for detecting fatty liver. However, the study had a small sample size, and future research should include larger, multi-center studies.

**Critical relevance statement:**

Ultrasound-derived fat fraction (UDFF) represents an objective technique characterized by high stability and reproducibility that accurately assesses liver fat content, which is suitable for the screening of metabolic dysfunction-associated steatotic liver disease (MASLD).

**Key Points:**

- Ultrasound-derived fat fraction (UDFF) obtained from the right lobe demonstrates strong repeatability and reproducibility.
- UDFF has good diagnostic performance in grading steatosis.
- UDFF is suitable for the screening of metabolic dysfunction-associated steatotic liver disease (MASLD).

**Graphical Abstract:**

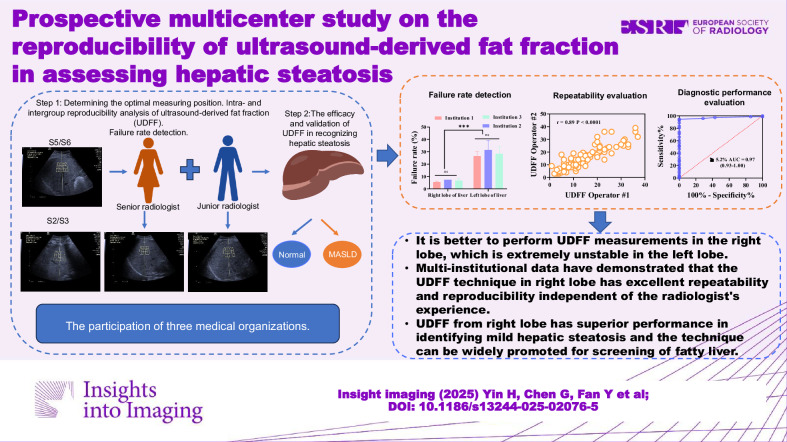

## Introduction

Non-alcoholic fatty liver disease (NAFLD) is the most prevalent liver disorder globally [1]. In 2023, the term metabolic dysfunction-associated steatotic liver disease (MASLD) was introduced as a more appropriate diagnostic label [2]. Without timely interventions targeting metabolic dysfunction, MASLD prevalence is projected to increase rapidly [3]. Therefore, early quantitative detection and personalized management of hepatic steatosis are essential for both the general population and high-risk groups.

Liver biopsy is considered the gold standard for diagnosing hepatic steatosis. However, it is invasive and carries risks such as pain, anxiety, bleeding, infection, and bile leakage, particularly in patients with coagulopathies [4]. MRI-based techniques, such as magnetic resonance proton density fat fraction (MRI-PDFF), offer a noninvasive and accurate alternative [5, 6], with strong correlation coefficients (> 0.9) with liver biopsy in fat quantification [7]. MRI-PDFF aligns well with histological grading (mild, moderate, severe) [8, 9]. Despite its diagnostic superiority, MRI-PDFF is costly and limited to major medical centers, hindering its broader use [10–12]. Conventional ultrasound is the most commonly used imaging modality for fatty liver disease [13], but its accuracy is highly dependent on the machine and operator [14]. Quantitative ultrasound techniques, including backscatter coefficients (BSC) and attenuation coefficients (AC), have shown promise in assessing hepatic steatosis [15]. Labyed et al proposed an integrated ultrasound-derived fat fraction (UDFF) model using linear least-squares analysis of AC and BSC to predict MRI-PDFF values [16].

UDFF offers several advantages over other noninvasive techniques [17]. Compared to serum biomarkers like the fatty liver index (FLI) and hepatic steatosis index (HSI), which are influenced by metabolic factors, UDFF provides morphological liver information that biomarkers cannot [18]. Unlike CT-based assessments, UDFF avoids radiation exposure, making it safer for children and pregnant women, and it is more cost-effective and accessible [19]. Compared to transient elastography (TE), such as FibroScan® using the controlled attenuation parameter (CAP), UDFF offers direct, quantitative liver fat assessment. CAP is semi-quantitative, affected by subcutaneous fat, and has higher failure rates in obese individuals. Moreover, TE lacks morphological liver information [18].

As a novel imaging biomarker, the clinical adoption of UDFF requires rigorous technical validation. Given ultrasound’s operator dependency [14, 20, 21], assessing the repeatability and reproducibility of UDFF among radiologists with different experience levels is critical [22]. This study aims to assess the repeatability and reproducibility of UDFF among operators with varying experience across different institutions, and to validate its diagnostic performance in detecting hepatic steatosis in patients suspected of MASLD.

## Materials and methods

### Study design

This prospective multicenter study was approved by the institutional ethics committee (B2023-215R) and registered at https://www.chictr.org.cn (ChiCTR2300069459). Written informed consent was obtained from all participants. From January to July 2023, individuals with clinical suspicion of MASLD were consecutively enrolled across three institutions for UDFF, liver ultrasound, and MRI-PDFF assessments. All UDFF examinations were performed using the ACUSON Sequoia system with a DAX probe. Each participant was scanned by two radiologists with different experience levels (≥ 10 years vs. < 3 years), who were blinded to clinical data. Participants fasted for at least 4 h, and MRI-PDFF was conducted within 14 days of UDFF. The study had two steps. First, we identified the optimal UDFF protocol by comparing failure rates between liver lobes, assessing inter-institutional repeatability, and determining the optimal number of measurements. Second, using MRI-PDFF as the reference, we evaluated the diagnostic performance of UDFF for hepatic steatosis.

### Sample size calculation

To ensure the reliability of the methodology, the sample size was calculated using the following formula:

$$n={\left(\frac{{Z}_{{1}^{-\alpha /2* \sqrt{p* (1-p)}}}}{\delta }\right)}^{2}$$

In the formula: *n* represents the sample size, α denotes the significance level, δ indicates the margin of error, ρ stands for sensitivity or specificity. For this study, we set *α* = 0.001 to minimize the risk of false positives. We selected *ρ* = 0.94 based on the specificity reported by Han et al [13], who developed a liver fat quantification method using raw RF data. Their study, using MRI-PDFF as the gold standard, reported a sensitivity of 0.97 and specificity of 0.94—values commonly used in fatty liver diagnostics. To ensure a conservative and adequately powered sample size, we used the specificity (0.94) for calculation, as it produces the largest sample size. Accounting for a 20% potential dropout rate, we estimated that at least 78 participants confirmed by MRI-PDFF would be required.

### Participant enrollment

The same inclusion and exclusion criteria were applied across all participating institutions. Inclusion criteria were: (a) age > 18 years; and (b) clinical suspicion of MASLD, defined by at least one of the following: (1) Imaging evidence of hepatic steatosis: (i) Ultrasound: Mild—hepatic echogenicity greater than the right renal cortex; Moderate—markedly increased echogenicity without perihepatic changes; Severe—obscured diaphragmatic echogenicity. (ii) MRI-PDFF grades: Grade 0 (< 5.2%), Grade 1 (5.2–11.3%), Grade 2 (11.3–17.1%), Grade 3 (≥ 17.1%) [23]. (2) Elevated serum biomarkers: abnormal AST, ALT, GGT, or dyslipidemia; (3) Metabolic risk factors: BMI ≥ 23 kg/m², type 2 diabetes, or other metabolic dysfunction [24]. Anthropometric and clinical data (age, sex, BMI, liver enzymes) were collected within two weeks before or after the ultrasound.

**Fig. 1:**
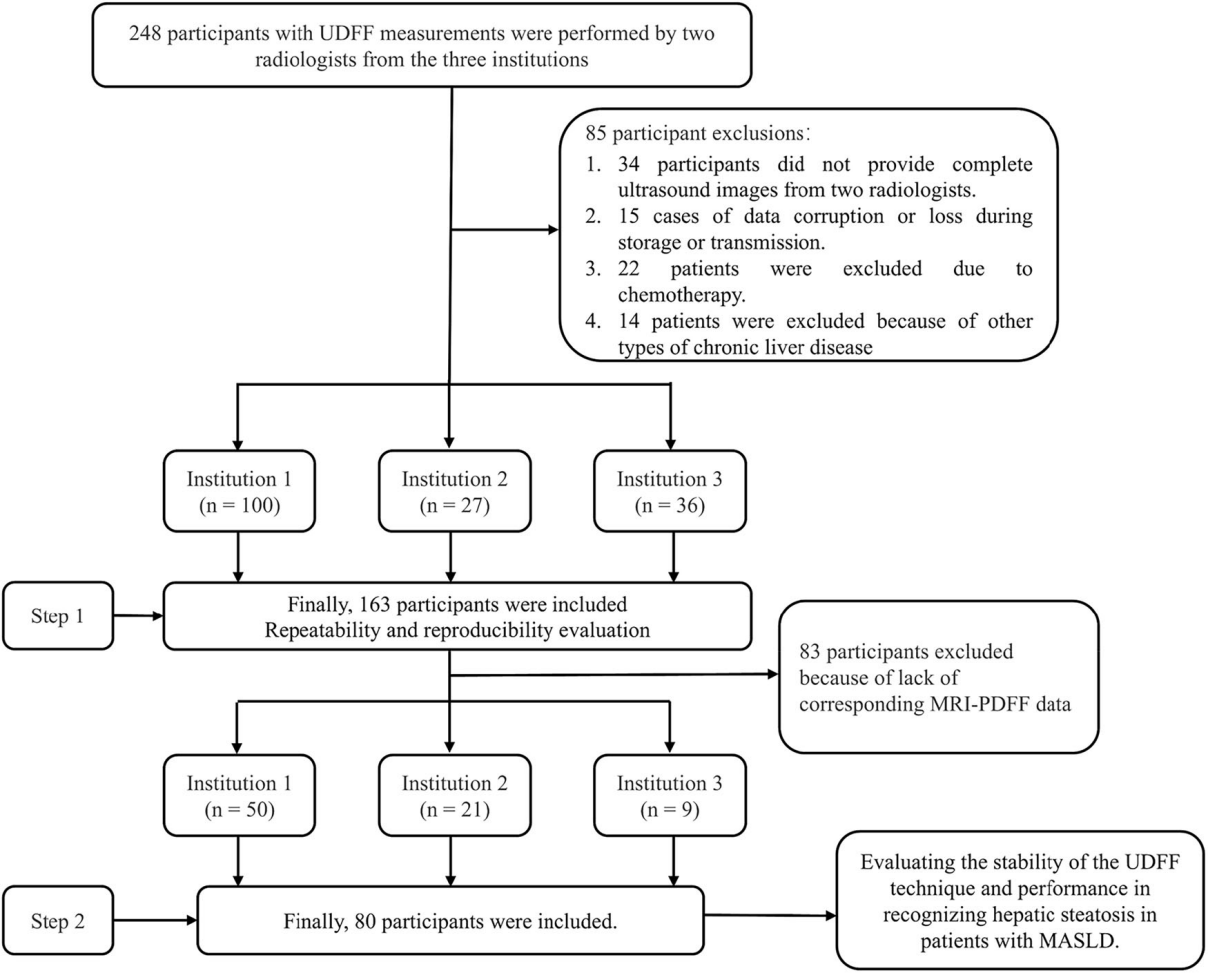
Flowchart of the patient enrollment procedure. UDFF, ultrasound-derived fat fraction; MRI-PDFF, magnetic resonance imaging-proton density fat fraction; MASLD, metabolism-associated fatty liver disease

In step one, 248 participants underwent ultrasound by two radiologists across three institutions. After exclusions—49 with incomplete data (34 lacked full scans; 15 had corrupted/lost files), 22 receiving systemic treatments, and 14 with other chronic liver diseases (e.g., viral or autoimmune hepatitis)—163 participants were included. In step two, 80 of the 163 had complete MRI-PDFF data and were included for further analysis to evaluate UDFF’s diagnostic performance (Fig. 1).

### UDFF measurements

All research centers used commercially available ultrasound equipment (ACUSON Sequoia US system; Siemens Healthineers) with a 5C1 convex probe (1.0–5.7 MHz) and a deep abdominal transducer (DAX). Each participant underwent two scans by radiologists with different experience, spaced 5–10 min apart. To assess UDFF stability and tissue heterogeneity in different liver lobes, a region of interest (ROI) was placed in segments S5/S6 of the right lobe and S2/S3 of the left lobe. Measurements were taken in the supine position using a right intercostal plane near the hepatic hilum, avoiding major vasculature and focal lesions. The probe was placed longitudinally and vertically under the xiphoid process, with the maximum section of the left liver lobe selected for UDFF measurement.

After conventional ultrasound, the UDFF mode was activated via the VT button. A 3 × 3 cm ROI was placed 1.5–2 cm below the liver capsule for accurate data acquisition. The “+” marker on the sampling line was aligned parallel to the liver capsule, and the patient held their breath for 1–3 s while images were captured. Five consecutive images were obtained, and the median UDFF value was used to estimate hepatic steatosis. Skin-to-capsule distance (SCD) was measured during the procedure. If the effective frame of 1.0 × 1.5 cm is less than 10, or if the final value is displayed as invalid, a UDFF measurement failure occurs. The failed measurement results will be retained for failure rate calculation but excluded from data analysis. Measurements will continue until five successful images are obtained.

UDFF measurements may be influenced by environmental factors like temperature, humidity, and noise, as well as equipment maintenance. To ensure consistency, all centers followed uniform equipment handling and calibration protocols. Instruments were kept at 22–25 °C in dry, well-ventilated areas, with daily calibration according to the manufacturer’s guidelines. Operators received standardized training and were assessed before data collection, minimizing measurement variability.

### MRI-PDFF procedure

All participating institutions used 1.5-T or 3.0-T MRI systems, equipped with either 18-channel or 32-channel coils. The patients were scanned in the supine position with a torso phased-array coil centered over the liver. MRI data acquisition was carried out using a VIBE (Volume Interpolated Breath-hold Examination) sequence in combination with DIXON technology for water-fat separation, which allowed two echoes to be acquired during a single breath-hold. The scanning coverage extended across the entire abdomen, from the liver dome to the iliac crest, to ensure full liver imaging. Key imaging parameters were as follows: axial scan plane, TR/TE = 5.49/2.46 ms, matrix size = 320 × 320, flip angle = 9°, field of view = 360 × 360 mm, slice thickness = 3 mm, and voxel size = 1.9 × 1.1 × 3.0 mm. The obtained DICOM images were then analyzed on dedicated post-processing workstations. Experienced radiologists (≥ 5 years of experience in abdominal MRI) manually placed regions of interest (ROIs) on the images to measure the proton density fat fraction (PDFF). Specifically, they delineated segments V, VI, and VIII, being careful to avoid large vessels, ducts, artifacts, and lesions. The PDFF values for each segment were averaged from three separate ROIs. For whole-liver PDFF, the fat fraction was calculated as the average of all eight liver segments. Median values were used in the analysis to reduce the influence of outliers, ensuring a more robust and accurate result.

### Statistical analysis

Statistical analyses were conducted using SPSS 26.0 and GraphPad Prism 8 software. The Kolmogorov-Smirnov test was used to assess data normality. Normally distributed data were presented as mean ± SD, while non-normally distributed data were presented as median (IQR). One-way ANOVA was used for multiple group comparisons. Pearson correlation and Bland-Altman analysis were performed to assess the agreement between UDFF values from different radiologists, with 95% limits of agreement (LOAs) [25]. Intraclass correlation coefficients (ICC) and 95% CIs were calculated using univariate two-way random effects ANOVA models, with ICC values interpreted as follows: 0–0.39 (poor), 0.40–0.59 (fair), 0.60–0.74 (good), and 0.75–1.0 (excellent) [14]. Univariate and multivariate binary logistic regression models analyzed factors influencing measurement failure in the left lobe. For UDFF1-UDFF2, univariate and multivariate linear regression included variables like BMI, skin-to-capsule distance, AST, ALT, and others. ROC curve analysis assessed the diagnostic value, with the optimal threshold determined by the Youden index. AUC comparisons used DeLong’s method [26]. A two-tailed test was applied, and *p* < 0.05 indicated statistical significance.

## Results

### Patient characteristics

In step one, 163 participants underwent UDFF measurements by two radiologists to assess repeatability and stability: 100 from Institution 1, 27 from Institution 2, and 36 from Institution 3. Among them, 89 (54.70%) were male and 74 (45.30%) were female, with a median age of 40 years (IQR: 33–55) and a mean BMI of 27.77 ± 6.40 kg/m² (IQR: 23.20–28.60). No significant differences were found in average UDFF values between the two radiologists across institutions. In step two, 80 participants (50 from Institution 1, 21 from Institution 2, 9 from Institution 3) underwent both UDFF and MRI-PDFF assessments to evaluate UDFF’s diagnostic performance. Of these, 73 (91.20%) were diagnosed with hepatic steatosis, while 7 (8.80%) had MRI-PDFF < 5.20%, indicating no steatosis (grade 0). Among steatosis cases, 13 were grade 1 (mild), 19 were grade 2 (moderate), and 41 were grade 3 (severe). Tables 1 and E1 summarized key demographic, clinical, laboratory, MRI-PDFF, and UDFF characteristics, along with overall and subgroup data by institution.

**Table 1:** Demographic, biochemical, histological, and imaging data of the participants

Variable	Value		
Sex	Female: 74 (45.30%)Male: 89 (54.70%)		
BMI (kg/m^2^)^a^	27.77 ± 6.4033 (BMI < 23), 130 (BMI ≥ 23)		
Age^b^	40 (33, 55)		
Skin-to-capsulate distance (SCD cm)^a^	2.27 ± 0.89		
Hemoglobin (Hb g/L)	141.88 ± 16.54		
White blood cell (WBC 10^9^/L)^a^	6.82 ± 2.27		
Platelets (PLT 10^9^/L)^a^	248.00 ± 66.23		
Alkaline phosphatase (ALP U/L)^a^	79.16 ± 26.99		
Serum creatinine (Cre μmol/L)^a^	66.51 ± 16.52		
Blood urea nitrogen (BUN mmol/L)^a^	5.09 ± 1.38		
Total cholesterol (TC mmol/L)^a^	4.98 ± 1.15		
Low-density lipoprotein (LDL mmol/L)^a^	3.14 ± 0.94		
Erythrocyte (RBC 10^9^/L)^b^	4.70 (4.40, 5.15)		
Neutrophil ratio (RGB %)^b^	53.20 (1.60, 63.05)		
Glutamic oxaloacetic transaminase (AST U/L)^b^	22.00 (17.00, 33.30)		
Glutamic pyruvic transaminase (ALT U/L)^b^	27.25 (17.15, 51.95)		
γ-glutamyl transpeptidase(γ-GGT U/L)^b^	32.00 (19.20, 56.50)		
Serum albumin (ALB g/L)^b^	45.50 (42.00, 48.20)		
Total bilirubin (TBil μmol/L)^b^	12.10 (9.40, 15.50)		
Direct bilirubin (DBil μmol/L)^b^	3.50 (2.55, 4.55)		
Triglycerides (TG mmol/L)^b^	1.43 (1.08, 2.20)		
High-density lipoprotein (HDL mmol/L)^b^	1.18 (1.18, 1.38)		
Fasting blood sugar (FPG mmol/L)^b^	5.50 (3.40, 9.45)		
MRI-PDFF	*N* (%)		
S0 MRI-PDFF < 5.2	6 (8.75%)^1^	1 (1.25%)^2^	0 (0%)^3^
S1: 5.2% ≤ MRI-PDFF < 11.3%	6 (8.75%)^1^	4 (5.00%)^2^	3 (3.75%)^3^
S2: 11.3% ≤ MRI-PDFF < 17.1%	13 (16.25%)^1^	5 (6.25%)^2^	1 (1.25%)^3^
S3: MRI-PDFF ≥ 17.1%	25 (31.25%)^1^	11 (13.75%)^2^	5 (6.25%)^3^

1, 2, and 3 refer to Institution 1, Institution 2, and Institution 3, respectively

*MRI-PDFF* magnetic resonance proton density fat fraction

^a^Mean with standard deviation

^b^Median with interquartile range

### Failure rate and analysis of influencing factors

As shown in Fig. E1, UDFF data from the left and right liver lobes were simultaneously collected by two radiologists. Failure rates were calculated for each institution. For the left lobe, failed measurements occurred in 24/100 and 29/100 participants at Institution 1, 7/27 and 10/27 at Institution 2, and 9/37 and 12/37 at Institution 3. Average left lobe failure rates were 26.5%, 31.5%, and 28.4%, respectively. For the right lobe, failures were much lower: 5/100 and 6/100 (Institution 1), 2/27 and 2/27 (Institution 2), and 2/37 and 3/37 (Institution 3), with average rates of 5.50%, 7.40%, and 6.60% (Fig. E1). Univariate binary logistic regression identified BMI, triglycerides, SCD, AST, ALT, and UDFF values as factors associated with left lobe failure. Multivariate regression confirmed UDFF value as an independent risk factor (Table 2).

**Table 2:** Factors associated with UDFF left liver lobe measurement failure results according to univariable and multivariable binary logistic regression analysis

Variables	Univariable analysis		Multivariable analysis	
	Coefficient (95% CI)	*p*-value	Coefficient (95% CI)	*p*-value
BMI	0.10 (1.05–1.16)	< 0.001***	−0.14 (0.76–1.73)	0.52
Age	−0.01 (0.97–1.01)	0.27		
SCD	1.19 (1.94–5.55)	< 0.001***	−2.03 (0.00–5.74)	0.29
WBC	−2.47 (1.04–1.50)	0.02*	0.25 (0.72–2.28)	0.41
Hb	0.05 (1.02–1.09)	< 0.001**	0.07 (0.96–1.19)	0.20
RBC	1.30 (1.63–8.31)	< 0.01**	0.12 (0.30–4.17)	0.86
RGB	0.01 (0.99–1.02)	0.26		
PLT	0.00 (1.00–1.01)	0.27		
Cre	0.01 (0.99–1.03)	0.47		
BUN	−0.37 (0.50–0.95)	0.02*	−0.71 (0.15–1.60)	0.23
TC	0.15 (0.87–1.56)	0.32		
LDL	−0.04 (0.67–1.39)	0.84		
HDL	−1.09 (0.11–1.04)	0.06		
AST	0.03 (1.02–1.05)	< 0.001***	0.01 (0.93–1.11)	0.79
ALT	0.02 (1.01–1.02)	< 0.001***	0.02 (0.98–1.05)	0.33
γ- GGT	0.00 (1.00–1.01)	0.20		
ALB	−0.00 (0.98–1.01)	0.74		
TG	0.45 (1.19–2.04)	< 0.001***	0.45 (0.72–3.46)	0.33
FPG	0.00 (0.96–1.05)	0.895		
UDFF	0.11 (1.08–1.16)	< 0.001***	0.29 (1.09–1.62)	< 0.01**

*BMI* body mass index, *SCD* skin-to-capsulate distance, *WBC* white blood cell, *Hb* hemoglobin, *RBC* erythrocyte, *RGB* neutrophil ratio, *PLT* platelets, *Cre* serum creatinine, *BUN* blood urea nitrogen, *LDL* low-density lipoprotein, *HDL* high-density lipoprotein, *TC* total cholesterol, *TG* triglycerides, *AST* glutamic oxaloacetic transaminase, *ALT* glutamic pyruvic transaminase, *γ-GGT* γ-glutamyl transpeptidase, *ALB* serum albumin, *FPG* fasting plasma glucose, *UDFF* ultrasound-derived fat fraction

* *p* < 0.05, ** *p* < 0.01, *** *p* < 0.001

### Repeatability and reproducibility of UDFF in the left liver lobe

A reproducibility analysis was performed on left liver lobe UDFF measurements by two radiologists across three centers. Moderate correlations were observed, with Pearson coefficients of 0.74 (95% CI: 0.60–0.84), 0.62 (95% CI: 0.21–0.85), and 0.78 (95% CI: 0.59–0.89). Bland-Altman analysis showed measurement differences of 1.30 (95% CI: −10.80 to 13.40), −0.38 (95% CI: −13.72 to 12.97), and 1.25 (95% CI: −9.80 to 12.30), respectively (Table E2). The ICCs for the left lobe UDFF were 0.85 (95% CI: 0.75–0.91), 0.76 (95% CI: 0.36–0.92), and 0.87 (95% CI: 0.74–0.94). Univariate and multivariate linear regression analyses were conducted to explore factors contributing to inter-operator differences. Univariate results identified age and SCD as significant factors influencing measurement discrepancies (Table E3).

### Inter-group repeatability and reproducibility of UDFF in the right liver lobe

**Fig. 2:**
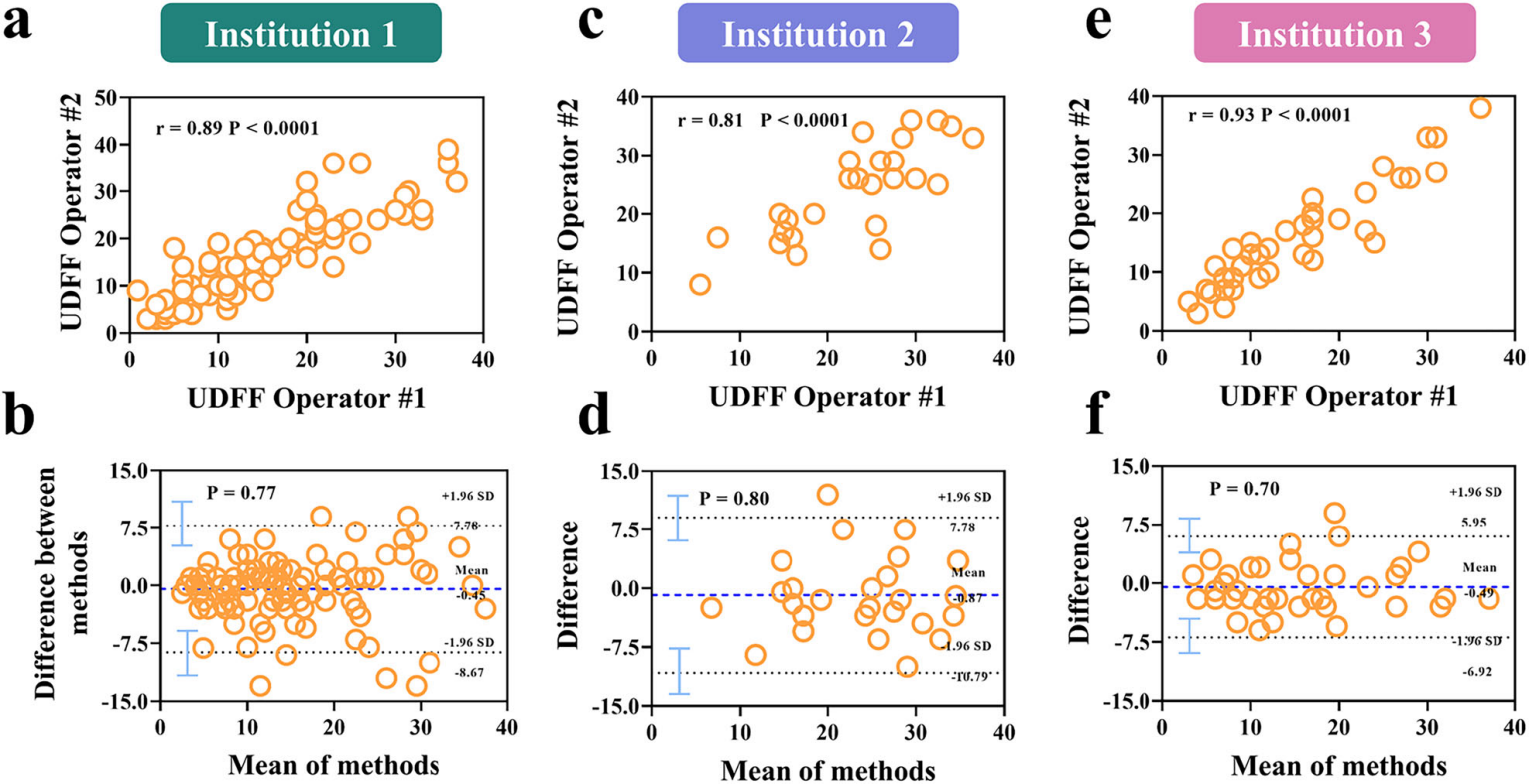
Inter-observer agreement of UDFF in three institutions. **a**, **c**, **e** UDFF obtained by two radiologists at three institutions showed strong correlation with Pearson correlation coefficients of 0.89 (*p* < 0.0001), 0.81 (*p* < 0.0001) and 0.93 (*p* < 0.0001), respectively. **b**, **d**, **f** Bland-Altman plots obtained from two radiologists at three institutions (*p* = 0.77, *p* = 0.80 and *p* = 0.70)

A reproducibility analysis was conducted on the UDFF measurements obtained from the right liver lobe by two radiologists across three centers. The results showed a significant correlation between the UDFF values obtained by the two operators at the right liver lobe across the three centers, with Pearson correlation coefficients of 0.89 (95% CI: 0.84–0.92, *p* < 0.0001), 0.81 (95% CI: 0.62–0.91, *p* < 0.0001), and 0.93 (95% CI: 0.87–0.96, *p* < 0.0001). Bland-Altman plots further indicated that there was no systematic overestimation or underestimation of UDFF assessments between the two operators at the three centers, and no trend of differences between the average scores. The differences in UDFF values between the two operators at the three centers were −0.45 (95% CI: −8.67 to 7.78), −0.87 (95% CI: −10.79 to 9.05), and −0.49 (95% CI: −6.92 to 5.95). Only eight patients across the three centers had scores outside the agreement range. Additionally, ICCs demonstrated good inter-operator consistency for UDFF values at the right liver lobe across all three centers, with ICCs of 0.94 (95% CI: 0.91–0.96), 0.89 (95% CI: 0.76–0.95), and 0.96 (95% CI: 0.93–0.98) (Fig. 2, Table 3).

**Table 3:** Interobserver reproducibility analysis results for UDFF in the right liver lobe at three institutions

Variables	Mean bias	LOA	ICC (95% CI)	Pearson r (95% CI)
Institution 1	−0.45 (*p* = 0.77)	−8.67 to 7.78	0.94 (0.91 to 0.96)	0.89 (0.84 to 0.92)
Institution 2	−0.87 (*p* = 0.80)	−10.79 to 9.05	0.89 (0.76 to 0.95)	0.81 (0.62 to 0.91)
Institution 3	−0.49 (*p* = 0.70)	−6.92 to 5.95	0.96 (0.93 to 0.98)	0.93 (0.87 to 0.96)

*BALA* Bland-Altman limits of agreement, *LOA* limits of agreement, *ICC* model used is the two-way random effects model, *CI* confidence interval

### Factors influencing inter-operator variability of UDFF in the right liver lobe

**Fig. 3:**
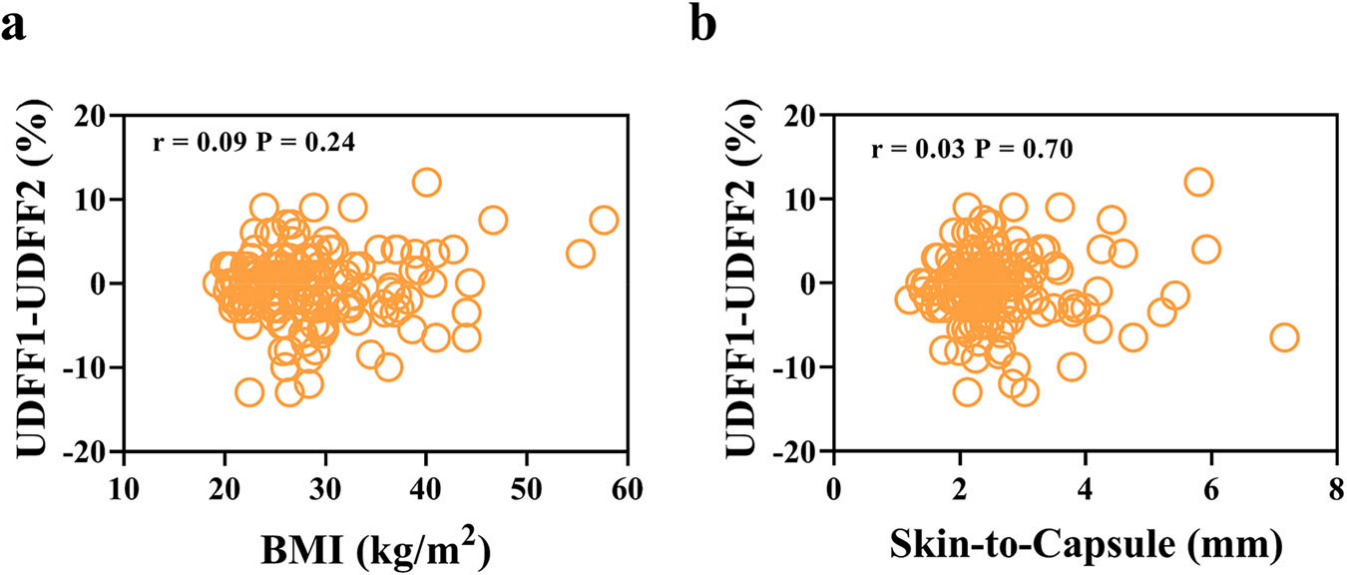
Analysis of factors influencing differences in UDFF values obtained by different radiologists. **a** Differences in UDFF obtained by different radiologists were not significantly correlated with BMI, with a Pearson’s correlation coefficient of 0.09, *p* = 0.24. **b** Differences in UDFF obtained by different radiologists were not significantly correlated with BMI, with a Pearson’s correlation coefficient of 0.03, *p* = 0.70

We conducted an analysis to explore the correlation between differences in UDFF values for the right liver lobe, obtained by different operators, and factors such as BMI or SCD. The results showed no significant correlation between UDFF values and either BMI or SCD. Specifically, the Pearson correlation coefficient between UDFF and BMI was 0.09 (*p* = 0.24), and between UDFF and SCD it was 0.03 (*p* = 0.70), indicating no significant relationship between these factors and the observed differences in UDFF measurements (Fig. 3). Subsequently, both univariate and multivariate linear regression analyses were performed to identify potential factors associated with the differences in UDFF measurements for the right liver lobe between operators. The analysis revealed no significant factors that could explain the variability in UDFF values for the right liver lobe. In the univariate analysis, the regression coefficient for the difference in UDFF values and SCD was 0.14 (−0.59 to 0.87), with *p* = 0.70. In the multivariate analysis, the regression coefficient for the difference in UDFF values and SCD was 0.63 (−4.31 to 17.38), with *p* = 0.18 (Table E4).

Furthermore, subgroup analysis revealed that regardless of whether BMI was < 23 or ≥ 23, the UDFF values obtained by different operators for the right liver lobe were highly correlated, with a Pearson correlation coefficient of 0.88. Bland-Altman analysis showed that the biases for BMI < 23 and BMI ≥ 23 were −0.74 (95% CI: −6.84 to 5.36) and −0.47 (95% CI: −9.05 to 8.10), respectively (Fig. E2). Additionally, further analysis was conducted to examine whether the differences between operators were related to the learning time. The results indicated that the UDFF differences for the right liver lobe were not associated with learning time. Even with a shorter learning period, stable and highly reproducible UDFF results could be obtained from the right liver lobe (Fig. E3).

### Intra-group repeatability and reproducibility of UDFF in the right liver lobe

The intra-observer repeatability of UDFF for liver parenchyma was excellent regardless of the number of acquisitions, with ICCs of 0.93–0.97 for two measurements, ICC of 0.94–0.97 for three measurements and ICC of 0.98–0.98 for five measurements at the three centers, respectively. The results indicated that UDFF provides the most consistent and reliable outcomes when measurements were repeated five times (ICC å 0.95). However, two repetitions were also sufficient to yield more consistent results (ICC > 0.90) (Table E5).

### Comparing the ability of UDFF obtained from the right and left liver lobes to identify mild or more hepatic steatosis

**Fig. 4:**
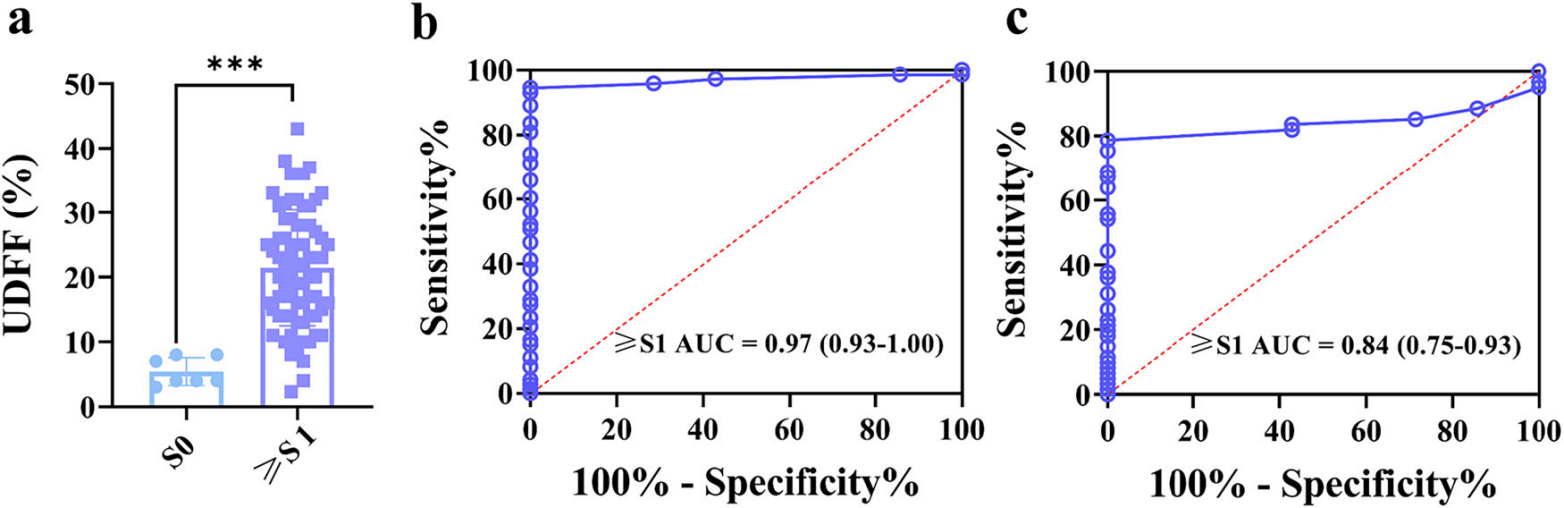
UDFF performance of hepatic steatosis ≥ S1. **a** UDFF scatterplot distribution between two groups with and without hepatic steatosis, using MRI-PDFF as a criterion (Right lobe of the liver). **b** Receiver operating characteristic curve used to detect hepatic steatosis ≥ S1, ROC = 0.97 (0.93–1.00). (Right lobe of the liver). **c** Receiver operating characteristic curve used to detect hepatic steatosis ≥ S1, ROC = 0.97 (0.93–1.00) (Left lobe of the liver)

A total of 80 patients underwent MRI-PDFF at three institutions. Figure 4 presented the scatterplot of UDFF distribution in the right liver lobe for steatosis and non-steatosis groups. The mean UDFF values for these groups were 5.43 ± 2.15% and 21.45 ± 8.96%, respectively (*p* < 0.001). UDFF measurements from the right lobe demonstrated a high diagnostic capability for hepatic steatosis (≥ S1), with an AUC of 0.97 (95% CI: 0.93–1.00) (Fig. 4). ROC analysis indicated that at a UDFF threshold of 8% in the right lobe, the sensitivity, specificity, positive predictive value (PPV), and negative predictive value (NPV) for diagnosing mild hepatic steatosis were 94.50%, 100.00%, 100.00%, and 63.60%, respectively (Table 4). When fixed values of 90% sensitivity and specificity, and 95% sensitivity and specificity were applied, the UDFF cutoff values in the right lobe were determined to be 9.77%, 7.65%, 7.65%, and 7.83%, respectively. In contrast, the AUC for identifying hepatic steatosis (≥ S1) using UDFF measured in the left lobe of the liver was 0.84 (95% CI: 0.75–0.93), which was significantly lower than the right lobe (Table 4).

**Table 4:** UDFF performance of hepatic steatosis ≥ S1

Variables	Cutoff value	Sensitivity (%)	Specificity (%)	PPV (%)	NPV (%)	AUC (95% CI)
Right lobe of liver	8.00	94.50	100.00	100.00	63.55	0.97 (0.91–1.00)
	9.77	90.00	100.00	100.00	48.95	/
	7.65	95.00	90.00	99.00	63.32	/
	7.65	95.00	90.00	99.06	63.31	/
	7.83	94.76	95.00	99.50	63.48	/
Left lobe of liver	10.50	78.69	100.0	100.00	35.00	0.84 (0.75–0.93)
	5.78	90.00	11.07	88.82	11.27	/
	5.01	95.00	0.18	89.24	0.18	/
	9.77	79.45	90.00	98.57	33.44	/
	9.88	79.07	95.00	99.28	34.24	/

Pairwise comparison of receiver operating characteristic curves. *p* = 0.01

*AUC* area under the receiver operating characteristic curve, *NPV* negative predictive value, *PPV* positive predictive value

When using a UDFF threshold of 8% in the right hepatic lobe to identify mild or greater hepatic steatosis, a total of four false-negative cases were observed, resulting in a false-negative rate of 5.4%, with no false-positive cases. Among borderline cases, only one participant with hepatic steatosis was misclassified as not having steatosis. In contrast, when applying a UDFF threshold of 10.50% in the left hepatic lobe, the number of false-negative cases increased to 15 (20.50%), while no false-positive cases were observed. However, within the borderline range, three participants with hepatic steatosis were misclassified (Table 5).

**Table 5:** Cross-tabulation between UDFF in the right liver lobe, UDFF in the left liver lobe, and MRI-derived PDFF of 5.2% or greater

Diagnostic test	MRI-PDFF ≥ 5.2%		Total (*n* = 80)
	Positive (*n* = 73)	Negative (*n* = 7)	
UDFF in the right liver lobe			
Positive	69 (94.50%)	0 (0.00%)	69 (86.30%)
Negative	4 (5.50%)	7 (100%)	11 (13.70%)
UDFF in the left liver lobe			
Positive	58 (79.50%)	0 (0.00%)	58 (72.50%)
Negative	15 (20.50%)	7 (100.00%)	22 (27.50%)

Data are number of participants, and data in parentheses are percentages. Detection of MRI protein density fat fraction (PDFF) of 5.2% or more indicating hepatic steatosis is defined as Positive. The ultrasound-derived fat fraction (UDFF) in the right liver lobe of more than 5% and the UDFF in the left liver lobe of more than 5% are defined as Positive

### Comparing the ability of UDFF obtained from the right and left liver lobes to identify moderate and severe hepatic steatosis

Further evaluation of the diagnostic performance of UDFF for moderate and severe hepatic steatosis revealed that UDFF measurements in the right hepatic lobe demonstrated superior diagnostic capability for hepatic steatosis (≥ S2) compared to those in the left lobe. The AUCs for the right and left lobes were 0.78 (95% CI: 0.68–0.87) and 0.69 (95% CI: 0.57–0.77), respectively (*p* = 0.04) (Fig. E4a, b). ROC analysis indicated that, at a UDFF threshold of 14% in the right lobe, the sensitivity, specificity, PPV, and NPV for diagnosing hepatic steatosis (≥ S2) were 79.70%, 66.70%, 87.05%, and 53.91%, respectively (Table E6). Similarly, at a UDFF threshold of 10% in the left lobe, the sensitivity, specificity, PPV, and NPV were 81.48%, 61.54%, 81.48%, and 61.53%, respectively (Table E6).

For hepatic steatosis (≥ S3), UDFF measurements in the right hepatic lobe also exhibited superior diagnostic performance, with AUC values of 0.82 (95% CI: 0.71–0.89) and 0.68 (95% CI: 0.56–0.78) for the right and left lobes, respectively (*p* = 0.02) (Fig. E4c, d). ROC analysis further demonstrated that, at a UDFF threshold of 22% in the right lobe, the sensitivity, specificity, PPV, and NPV for diagnosing severe hepatic steatosis (≥ S3) were 68.29%, 84.62%, 84.09%, and 72.16%, respectively (Table E6). Additionally, at a UDFF threshold of 10.0% in the right lobe, the sensitivity, specificity, PPV, and NPV for diagnosing severe hepatic steatosis were 90.00%, 46.00%, 50.00%, and 88.46%, respectively (Table E6).

## Discussion

Over the past decade, quantitative ultrasound has emerged as a promising noninvasive tool for assessing hepatic steatosis, fibrosis, and therapeutic response in chronic liver disease, including high-risk NASH [27]. Hepatic steatosis, the earliest and most critical histological manifestation of MASLD, contributes significantly to liver-related morbidity and mortality [28]. If left untreated, MASLD progresses from an asymptomatic phase to cirrhosis and potentially hepatocellular carcinoma [29]. As a new technique, UDFF requires validation of its repeatability and reproducibility before widespread clinical use.

This study first evaluated the technical reliability of UDFF at three tertiary institutions. The right hepatic lobe showed the best performance, with a failure rate of only 5.5–7.4%, whereas the left lobe showed higher failure rates (26.5–31.5%). Univariate logistic regression indicated that higher BMI, triglyceride levels, subcutaneous fat thickness (SCD), and UDFF values were associated with failure in the left lobe. Multivariate analysis confirmed UDFF as an independent predictor. These findings suggest that subcutaneous fat, steatosis severity, and BMI increase the likelihood of left-lobe measurement failure. Additionally, several other factors might contribute to the higher failure rate in the left lobe: (1) Anatomical position and structural interference: The presence of surrounding organs, such as the beating heart and intragastric gas, may interfere with measurements. Moreover, the smaller volume and thinner structure of the left lobe may result in insufficient probe contact, compromising measurement stability and reliability [30]. (2) Acoustic window limitations: The acoustic window for the left lobe is often obstructed by ribs, pulmonary air, or gastrointestinal gas [30]. (3) Technical challenges in operation: Controlling the probe angle and pressure is more difficult when measuring the left lobe [31].

Despite these limitations, UDFF measurement in the right lobe was stable and consistent. Pearson correlation showed high agreement between senior and junior radiologists for right-lobe measurements, while left-lobe results were more influenced by factors such as SCD and age. These findings reinforce the recommendation to use the right hepatic lobe for UDFF. Intra-operator reproducibility was excellent for right-lobe UDFF, with ICCs > 0.92 for at least two measurements and > 0.97 for five, supporting UDFF’s utility in quantifying steatosis. Thus, a minimum of two and ideally five right-lobe measurements is recommended in clinical practice.

**Fig. 5:**
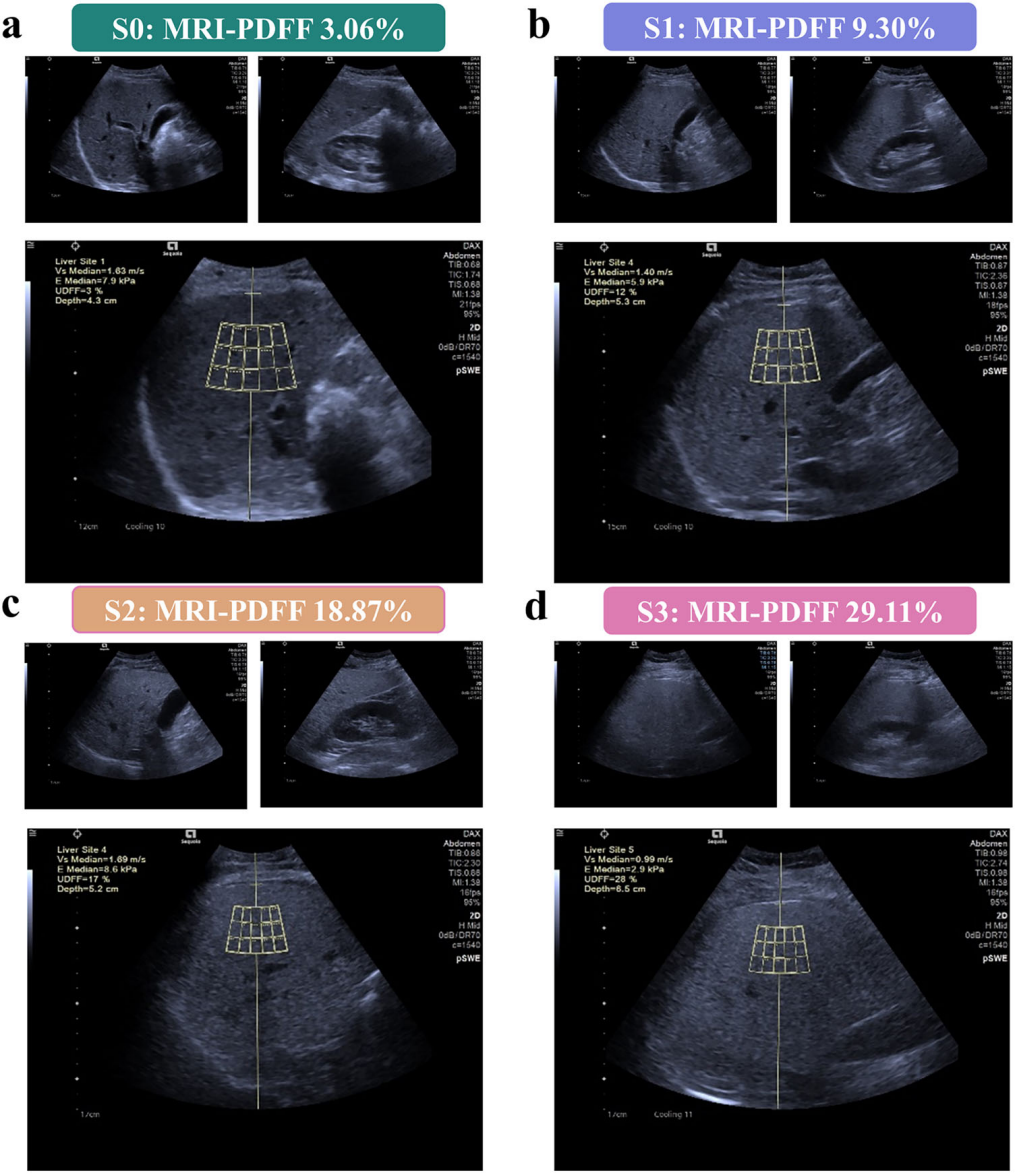
UDFF images of representative 4 participants with hepatic steatosis graded according to MRI-PDFF. **a** For a 57-year-old female participant without hepatic steatosis, the UDFF image (UDFF = 4.3%) with an MRI-PDFF of 3.06%. **b** For a 37-year-old male participant with mild hepatic steatosis, the UDFF image (UDFF = 12%) with an MRI-PDFF of 9.30%. **c** For a 39-year-old male participant with moderate hepatic steatosis, the UDFF image (UDFF = 17%) with an MRI-PDFF of 18.87%. **d** For a 36-year-old male participant with severe hepatic steatosis, the UDFF image (UDFF = 28%) with an MRI-PDFF of 29.11%

In step two, the diagnostic performance of UDFF was evaluated against MRI-PDFF (≥ 5.2%) for detecting mild hepatic steatosis. The results indicated that UDFF derived from the right lobe of the liver exhibited superior diagnostic performance in detecting mild hepatic steatosis (Fig. 5). Right-lobe UDFF yielded superior results (AUC = 0.97 vs. 0.84 in left lobe), outperforming a previous study by De Robertis et al (AUC = 0.75), likely due to the more balanced distribution of steatosis severity in our cohort (S0 = 7, S1 = 13, S2 = 19, S3 = 41) [32]. Right-lobe UDFF also showed a lower false-negative rate. We also evaluated the ability of UDFF to assess different degrees of hepatic steatosis. For moderate-to-severe (MRI-PDFF ≥ 11.3%) and severe steatosis (≥ 17.1%), right-lobe UDFF again outperformed the left. AUCs for moderate-to-severe steatosis were 0.78 (right) vs. 0.69 (left) (*p* = 0.04), and for severe steatosis, 0.82 (right) vs. 0.68 (left) (*p* = 0.02).

Compared with other ultrasound-based methods like CAP, ATI, UGAP, TAI, and BSC, UDFF demonstrated competitive or superior accuracy, especially using MRI-PDFF as a reference (standard AUCs: 0.84–0.95) [33–36]. UDFF offers a major advantage: its reference standard, MRI-PDFF, provides a quantifiable steatosis percentage, making results more intuitive for clinicians and patients. It showed a 100% positive predictive value in diagnosing steatosis, which may reduce screening failures in MASLD trials and help identify suitable trial candidates [37]. UDFF is also cost-effective compared to MRI-PDFF. Ultrasound equipment is significantly cheaper and more accessible in primary care or rural settings, making UDFF more suitable for large-scale or repeated screening. As a result, UDFF has the potential to become a widely available, economical, and reliable method for early MASLD detection and monitoring [38, 39].

Several limitations of this study should be noted. First, the relatively small number of patients with MRI-PDFF reduced the robustness of diagnostic performance, especially for advanced steatosis grades. Second, this study did not assess inflammation or fibrosis associated with MASLD. Third, liver biopsy was not used, and further studies are needed to validate UDFF against histopathology. Fourth, the study lacked long-term follow-up data to assess test–retest reliability and did not consider technical artifacts or ROI placement factors.

In conclusion, the study indicated that obtaining UDFF from the right liver lobe is reliable and effective for identifying hepatic steatosis, irrespective of the radiologist’s experience. This technique is recommended for the quantification and grading of hepatic steatosis. UDFF holds significant potential as a new noninvasive quantitative diagnosis tool, but further research with larger and more diverse samples and in patients with moderate-to-severe steatosis, inflammation, or fibrosis is needed to further expand its clinical applicability.

## Supplementary information

ELECTRONIC SUPPLEMENTARY MATERIAL

## Data Availability

The datasets used or analyzed during the current study are available from the corresponding author on reasonable request.

## Abbreviations

AUC: Area under the curve
BMI: Body mass index
CI: Confidence interval
MASLD: Metabolic dysfunction-associated steatotic liver disease
MRI-PDFF: Magnetic resonance proton density fat fraction
NAFLD: Nonalcoholic fatty liver disease
NPV: Negative predictive value
PPV: Positive predictive value
ROC: Receiver operating characteristic curve
UDFF: Ultrasound-derived fat fraction
