# Supplementary material for: Prospective multicenter study on the reproducibility of ultrasound-derived fat fraction in assessing hepatic steatosis
Source: Insights Imaging. 2025 Nov 4;16:243. doi: 10.1186/s13244-025-02076-5 (PMC12586825; doi:10.1186/s13244-025-02076-5)
Supplement: Supplementary file 1 — ELECTRONIC SUPPLEMENTARY MATERIAL [file 13244_2025_2076_MOESM1_ESM.pdf]

Prospective Multicenter Study on the Reproducibility of Ultrasound-Derived Fat Fraction in Assessing Hepatic Steatosis

ELECTRONIC SUPPLEMENTARY MATERIAL

Table E1. The mean UDFF values were acquired from the right lobe of the liver by radiologists with varying levels of experience in five institutions.

Variable	Variable	Mean Value (Right)	Mean Value (Left)
Institution 1 (N = 100)	UDFF-1 (%)[a]	13.56 ± 8.96	16.46 ± 8.61
	UDFF-2 (%)[a]	14.57 ± 8.81	15.16 ± 7.80
Institution 2 (N = 27)	UDFF-1 (%)[a]	23.46 ± 8.26	21.71 ± 8.27
	UDFF-2 (%)[a]	24.33 ± 8.01	22.09 ± 7.43
Institution 3 (N = 36)	UDFF-1 (%)[a]	15.01± 8.56	19.55 ± 8.41
	UDFF-2 (%)[a]	15.91 ± 8.69	18.30 ± 8.64

[a] Mean value provided with standard deviations; UDFF: ultrasound-derived fat fraction .

UDFF-1: UDFF values obtained by the senior radiologist in the right lobe of the liver.

UDFF-2: UDFF values obtained by the junior radiologist in the right lobe of the liver.

The One-Way ANOVA analysis showed no significant difference in UDFF measurements between Institution 1 and Institution 3, while there was a significant difference in UDFF measurements between these institutions and Institution 2 ($P < 0.01$). Within Institution 2, however, there was no significant difference in UDFF measurements.

Table E2. Interobserver reproducibility analysis results for UDFF in left liver lobe at three institutions.

Variables	Mean bias	LOA	ICC (95% CI)	Pearson r (95% CI)
Institution 1	1.30 (*P* = 0.49)	-10.80 to 13.40	0.85 (0.75 to 0.91)	0.74 (0.60 to 0.84)
Institution 2	-0.38 (*P* = 0.60)	-13.72 to 12.97	0.76 (0.36 to 0.92)	0.62 (0.21 to 0.85)
Institution 3	1.25 (*P* = 0.82)	-9.80 to 12.30	0.87 (0.74 to 0.94)	0.78 (0.59 to 0.89)

BALA: Bland-Altman limits of agreement; LOA = limits of agreement; ICC model used is the two-way random effects model; CI: confidence interval;

Table E3. Factors associated with the differences in UDFF left liver lobe measurements according to univariable and multivariable linear regression analysis.

Variables	Univariable Analysis		Multivariable Analysis	
	Coefficient (95% CI)	P	Coefficient (95% CI)	P
**BMI**	-0.08(-0.27-0.12)	0.42		
**Age**	0.09 (0.01-0.17)	0.02*	0.08 (-0.01-0.16)	0.07
**SCD**	-1.32 (-2.52-1.12)	0.03*	-0.98 (-2.22-0.25)	0.12
**WBC**	0.32 (-0.80-0.49)	0.62		
**Hb**	0.05 (-0.16-0.05)	0.30		
**RBC**	-2.00 (-4.32-0.32)	0.09		
**RGB**	-0.10 (-0.09-0.08)	0.83		
**PLT**	0.02 (-0.00-0.05)	0.07		
**Cre**	-0.07 (-0.17-0.04)	0.20		
**BUN**	-0.18 (-1.54-1.18)	0.79		
**TC**	-0.14 (-1.51-1.22)	0.84		
**LDL**	0.17 (-1.84-2.18)	0.87		
**HDL**	2.96 (-0.17-6.10)	0.06		
**AST**	0.02 (-0.02-0.06)	0.40		
**ALT**	0.01 (-0.02-0.04)	0.42		
**γ- GGT**	0.01 (-0.01-0.03)	0.58		
**ALB**	-0.06 (-0.24-0.12)	0.52		
**TG**	-0.33 (-1.28-0.61)	0.48		
**FPG**	0.08 (-0.06-0.22)	0.28		

BMI: body mass index, SCD: Skin-to-capsulate distance, WBC: White blood cell, Hb: Haemoglobin, RBC: Erythrocyte, RGB: Neutrophil Ratio, PLT: Platelets, Cre: Serum creatinine, BUN: Blood Urea Nitrogen, LDL: Low-density lipoprotein, HDL: High-density lipoprotein, TC: Total cholesterol, TG: Triglycerides, AST: Glutamic oxaloacetic transaminase, ALT: Glutamic pyruvic transaminase, γ-GGT: γ-glutamyl transpeptidase, ALB: Serum albumin, FPG: Fasting plasma glucose.

*P<0.05, **P<0.01, ***P<0.001.

Table E4. Factors associated with the differences in UDFF right liver lobe measurements according to univariable and multivariable linear regression analysis.

Variables	Univariable Analysis		Multivariable Analysis	
	Coefficient (95% CI)	P	Coefficient (95% CI)	P
**BMI**	0.06 (-0.04-0.16)	0.24	0.16 (-1.15-1.48)	0.32
**Age**	-0.00 (-0.05-0.04)	0.93	0.16 (-0.35-0.67)	0.46
**SCD**	0.14 (-0.59-0.87)	0.70	0.63 (-4.31-17.38)	0.18
**WBC**	-0.05 (-0.47-0.36)	0.80	1.71 (-2.01-5.43)	0.29
**Hb**	0.02 (-0.04-0.08)	0.58	0.27 (-0.12-0.66)	0.14
**RBC**	0.24 (-1.18-1.67)	0.74	9.26 (-22.09-25.51)	0.83
**RGB**	0.01 (-0.04-0.07)	0.57	-0.50 (-1.34-0.34)	0.19
**PLT**	-0.00 (-0.02-0.01)	0.83	0.03 (0.01-0.05)	<0.01**
**Cre**	0.00 (-0.05-0.06)	0.89	-0.01 (-0.58-0.56)	0.98
**BUN**	-0.04 (-0.72-0.65)	0.92	4.12 (-2.16-10.40)	0.15
**TC**	0.33 (-0.50-1.16)	0.43	-5.14 (-26.62-16.34)	0.57
**LDL**	-0.69 (-1.44-0.06)	0.07	1.75 (-23.32-26.83)	0.33
**HDL**	-0.77 (-2.53-1.00)	0.39	9.35 (-12.92-31.63)	0.86
**AST**	-0.02 (-0.04-0.01)	0.13	-0.20 (-0.54-0.14)	0.20
**ALT**	-0.00 (-0.02-0.01)	0.85	0.03 (-0.25-0.31)	0.81
**γ- GGT**	-0.01 (-0.01-0.00)	0.17	0.03 (-0.17-0.22)	0.74
**ALB**	0.04 (-0.08-0.16)	0.49	-0.34 (-2.02-1.33)	0.62
**TG**	0.25 (-0.69-0.32)	0.47	-1.38 (-6.69-3.93)	0.53
**FPG**	-0.06 (-0.15-0.02)	0.16	0.63 (-2.22-3.47)	0.18

BMI: body mass index, SCD: Skin-to-capsulate distance, WBC: White blood cell, Hb: Haemoglobin, RBC: Erythrocyte, RGB: Neutrophil Ratio, PLT: Platelets, Cre: Serum creatinine, BUN: Blood Urea Nitrogen, LDL: Low-density lipoprotein, HDL: High-density lipoprotein, TC: Total cholesterol, TG: Triglycerides, AST: Glutamic oxaloacetic transaminase, ALT: Glutamic pyruvic transaminase, γ-GGT: γ-glutamyl transpeptidase, ALB: Serum albumin, FPG: Fasting plasma glucose.

*P<0.05, **P<0.01, ***P<0.001.

Table E5. Intraobserver reproducibility was analyzed for three institutions based on the mean UDFF values from multiple collections (N = 2, 3, 4, and 5).

Variables	Institution 1: ICC (95% CI)	Institution 2: ICC (95% CI)	Institution 3: ICC (95% CI)
N = 2	0.97 (0.96-0.98)	0.95 (0.92-0.97)	0.93 (0.83-0.97)
N = 3	0.97 (0.97-0.98)	0.97 (0.95-0.98)	0.95 (0.91-0.98)
N = 5	0.98 (0.98-0.99)	0.98 (0.97-0.99)	0.98 (0.96-0.99)

ICCs were calculated using a single-unit, absolute agreement two-way mixed analysis of variance (ANOVA) model. CI: confidence interval; ICC: intraclass correlation coefficien.

Table E6. UDFF performance of hepatic steatosis ≥S2 or ≥S3.

Variables		Cut-off value	Sensitivity (%)	Specificity (%)	PPV (%)	NPV (%)	AUC (95% CI)
≥S2	Right	14.00	79.70	66.70	87.05	53.91	0.78 (0.68-0.87)*
	Left	10.00	81.48	61.54	81.48	61.53	0.69 (0.57-0.77)
≥S3	Right	22.00	68.29	84.62	84.09	72.16	0.82 (0.71-0.89) *
	Left	10.00	90.00	46.00	50.00	88.46	0.68 (0.56-0.78)

AUC: area under the receiver operating characteristic curve; NPV: negative predictive value: PPV: positive predictive value. Pairwise comparison of receiver operating characteristic curves. $P < 0.05$

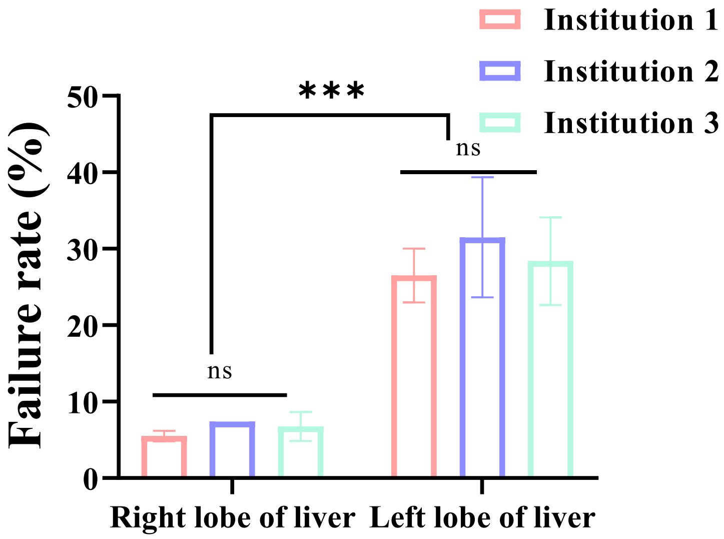

Figure E1. UDFF failure rates were obtained in the left and right lobes of liver at three institutions.

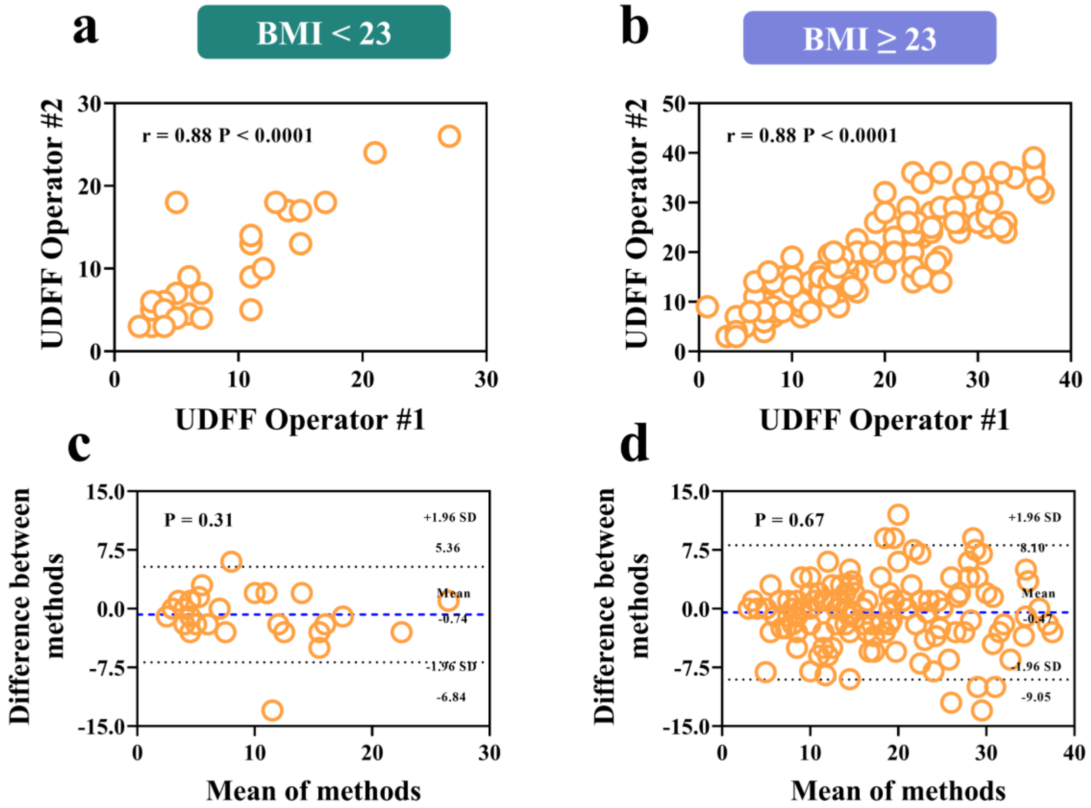

Figure E2. Interobserver agreement of UDFF measurements stratified by BMI across three institutions. (a) UDFF obtained by two radiologists at three institutions showed strong correlation with Pearson correlation coefficients of 0.88 (P < 0.0001). (b) Similarly, UDFF measurements in participants with BMI ≥23 kg/m² showed excellent interobserver agreement, with Pearson correlation coefficients of 0.88 (P < 0.0001). (c) Bland-Altman plots comparing UDFF measurements between two radiologists in participants with BMI <23 kg/m² across three institutions (P = 0.31). (d) Bland-Altman analysis of UDFF measurements in participants with BMI ≥23 kg/m² showed comparable agreement (P = 0.67).

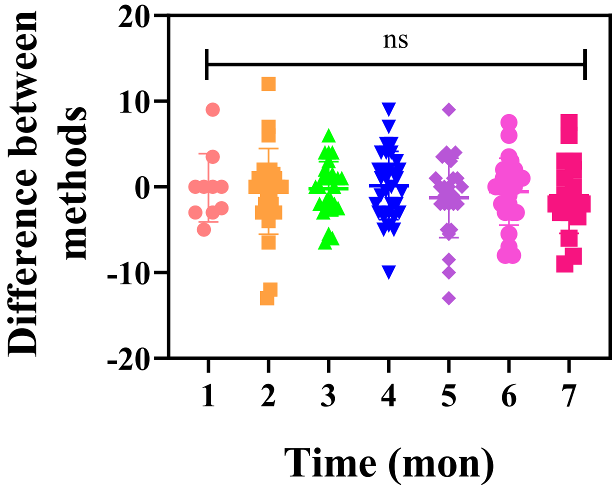

Figure E3. The changes in the UDFF measurement differences for the right liver lobe by the two radiologists with the extension of learning time.

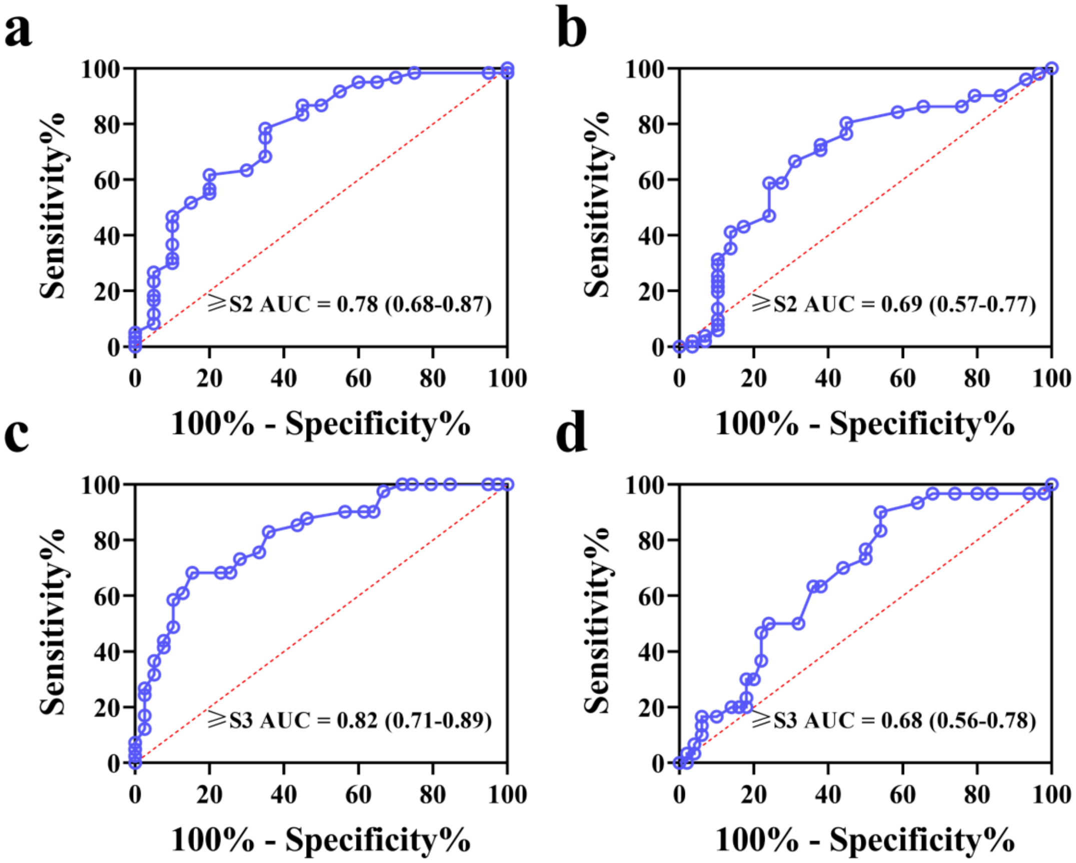

Figure E4. UDFF performance of hepatic steatosis ≥S2 or =S3. (a) Receiver operating characteristic curve used to detect hepatic steatosis ≥S2, ROC = 0.78 (0.68-0.87). (Right lobe of the liver). (b) Receiver operating characteristic curve used to detect hepatic steatosis ≥S2, ROC = 0.69 (0.57-0.77). (Left lobe of the liver). (c) Receiver operating characteristic curve used to detect hepatic steatosis =S2, ROC = 0.82 (0.71-0.89). (Right lobe of the liver). (d) Receiver operating characteristic curve used to detect hepatic steatosis =S3, ROC = 0.68 (0.56-0.78). (Left lobe of the liver).
